# Complex coding of endogenous siRNA, transcriptional silencing and H3K9 methylation on native targets of germline nuclear RNAi in *C. elegans*

**DOI:** 10.1186/1471-2164-15-1157

**Published:** 2014-12-22

**Authors:** Julie Zhouli Ni, Esteban Chen, Sam Guoping Gu

**Affiliations:** Department of Molecular Biology and Biochemistry, Rutgers the State University of New Jersey, Nelson Labs A125, 604 Allison Road, Piscataway, NJ 08854 USA

**Keywords:** Nuclear RNAi, piRNA, Endo-siRNA, Transcriptional silencing, Heterochromatin, Argonaute protein, Retrotransposon silencing, Germline, ChIP-seq, Pre-mRNA-seq

## Abstract

**Background:**

Small RNA-guided transcriptional silencing (nuclear RNAi) is fundamental to genome integrity and epigenetic inheritance. Despite recent progress in identifying the capability and genetic requirements for nuclear RNAi in *Caenorhabditis elegans*, the natural targets and cellular functions of nuclear RNAi remain elusive*.*

**Methods:**

To resolve this gap, we coordinately examined the genome-wide profiles of transcription, histone H3 lysine 9 methylation (H3K9me) and endogenous siRNAs of a germline nuclear Argonaute (*hrde-1*/*wago-9*) mutant and identified regions on which transcription activity is markedly increased and/or H3K9me level is markedly decreased relative to wild type animals.

**Results:**

Our data revealed a distinct set of native targets of germline nuclear RNAi, with the H3K9me response exhibiting both overlapping and non-overlapping distribution with the transcriptional silencing response. Interestingly LTR retrotransposons, but not DNA transposons, are highly enriched in the targets of germline nuclear RNAi. The genomic distribution of the native targets is highly constrained, with >99% of the identified targets present in five autosomes but not in the sex chromosome. By contrast, HRDE-1*-*associated small RNAs correspond to all chromosomes. In addition, we found that the piRNA pathway is not required for germline nuclear RNAi activity on native targets.

**Conclusion:**

Germline nuclear RNAi in *C. elegans* is required to silence retrotransposons but not DNA transposon. Transcriptional silencing and H3K9me can occur independently of each other on the native targets of nuclear RNAi in *C. elegans*. Our results rule out a simple model in which nuclear Argonaute protein-associated-small RNAs are sufficient to trigger germline nuclear RNAi responses. In addition, the piRNA pathway and germline nuclear RNAi are specialized to target different types of foreign genetic elements for genome surveillance in *C. elegans.*

**Electronic supplementary material:**

The online version of this article (doi:10.1186/1471-2164-15-1157) contains supplementary material, which is available to authorized users.

## Background

RNA interference (RNAi) was originally discovered as a biochemical pathway triggered by double-stranded RNA (dsRNA) that leads to degradation of target mRNA with the corresponding sequence [1, 2]. RNAi is initiated by cutting dsRNA into small RNAs of 20–30 nucleotides (called small interfering RNAs, or siRNAs) by the RNase III-like enzyme dicer [3]. The resulting siRNAs are loaded into the highly conserved Argonaute (AGO) family of RNA-binding proteins, defined by the PIWI and PAZ domains [4]. Target mRNAs, through their base-pairing interactions with siRNAs, can then be degraded by the endonucleolytic activity (‘slicer’) of Argonaute proteins [5–7]. In plants, fungi, and *C. elegans*, dicer-produced siRNAs (named primary siRNAs) can also trigger *de novo* synthesis of additional small RNAs (called “secondary siRNAs”) through recruitment of RNA-directed RNA polymerases (RdRPs) that use the mature target mRNA as a template [8–10]. Endogenous small RNAs that are antisense to transcripts also exist in a variety of eukaryotic species. These so-called endo-siRNAs modulate a diverse set of cellular processes, such as gene expression, genome surveillance, chromosome transmission, and behavior adaptation [11–19].

In addition to mRNA degradation, RNAi has been found to function in the nucleus of plants and *Schizosaccharomyces pombe*. Nuclear siRNAs in these organisms guide Argonaute proteins and other protein factors to silence transcription and form a repressive chromatin state on target genes [reviewed in [20–23]]. This process has been termed “nuclear RNAi” (reviewed in [24]), as distinguished from the RNA-triggered mRNA degradation mechanism, referred to as “classical RNAi”.

Recent studies in *Drosophila* and *C. elegans* demonstrated that nuclear RNAi mechanisms play crucial functions in animals as well. In *Drosophila*, three PIWI subfamily members of Argonaute proteins, *AGO3, Aubergine,* and *Piwi*, in concert with PIWI-associated small RNAs (piRNAs) guide molecules, silence transposons in both somatic and germ cells [25, 26]. Gene silencing mediated by *AGO3* and *Aubergine* occurs at the post-transcriptional level [26], while the *Piwi* protein leads to transcriptional silencing and heterochromatin response on its target transposons [27–29].

Nuclear RNAi in *C. elegans* was initially suggested by the reduction of target transcripts in the nucleus when animals were treated with exogenous dsRNA (exo-dsRNA) [30]. Recent studies using genetics, biochemistry, and whole-genome approaches demonstrated that exo-dsRNA triggers transcriptional silencing as well as H3K9 methylation (H3K9me), a histone mark associated with the repressive chromatin state [31–34], on the target loci [35–39]. A diverse set of protein-coding genes was found to be susceptible to exo-dsRNA-induced nuclear RNAi [36, 39]. These features, combined with powerful genetics*,* make *C. elegans* a highly attractive system to study the mechanisms of nuclear RNAi. Several genes, including *nrde-1, −2, −3, and −4,* have been recently identified for their essential roles in the nuclear RNAi pathway [35–37].

Nuclear RNAi in *C. elegans* is a *bona fide* epigenetic process. For a number of germline-expressed genes, the exo-dsRNA-induced silencing state, as well as target-specific H3K9 trimethylation, can last for several generations [39]. The epigenetic inheritance of the RNAi effects is dependent on the nuclear RNAi pathway. Recent studies identified HRDE-1, a nucleus-localized Argonaute protein, for its essential role in heritable RNAi [38, 40, 41]. HRDE-1 is one of several worm-specific AGO proteins and was initially named as *WAGO-9* [42]. The name HRDE-1 is used in this work to reflect the *h*eritable *R*NAi-*de*ficient phenotype of the mutant. HRDE-1 is highly expressed in the *C. elegans* germline and appears to be absent in somatic cells, so it is considered as a germline-specific nuclear Argonaute protein. In contrast, another nuclear Argonaute protein NRDE-3 appears to work solely in the somatic cells. Single-mutant animals *hrde-1*, *nrde-1, nrde-2,* and *nrde-4* all exhibit reduced viability of germ cells at elevated temperatures, indicating a crucial function mediated by nuclear RNAi in germline development [38].

Despite recent progress in identifying the genetic requirements for nuclear RNAi, its cellular functions in *C. elegans* remain elusive. Previous studies have shown that a large population of endo-siRNAs are loaded into the germline nuclear Argonaute protein HRDE-1 [38], suggesting that many regions of the genome can potentially be targeted by nuclear RNAi. In addition, mutations in *nrde-2* and *nrde-4* lead to loss of H3K9 trimethylation in distinct loci [38]. While these studies clearly indicate important roles of nuclear RNAi in regulating chromatin structures, the global impact of nuclear RNAi on gene transcription has not been examined. Resolving this critical gap will identify the native targets of nuclear RNAi and delineate its endogenous functions in regulating gene expression and epigenetic response during animal development. To this end, we combined genetic, biochemical, and whole-genome computational approaches to identify and characterize transcriptional silencing events that are dependent on the germline nuclear Argonaute protein HRDE-1*.*

## Results and discussion

### The germline nuclear Argonaute protein HRDE-1 is required for the exclusion of RNA Polymerase II (Pol II) in a distinct set of genomic regions

To identify native targets of germline nuclear RNAi, we performed Pol II ChIP-seq using *C. elegans* wild-type populations and animals carrying a *hrde-1* mutation. For this work, we used the *hrde-1*(*tm1200*) mutant allele, which has a 376-bp deletion in exon 3 and results in a premature stop codon before the essential PAZ and PIWI domains. This allele was also used in previous studies as a loss-of-function mutation [38, 40, 41]. To enrich for germline material, we used young adult hermaphrodites for both samples, in which germline contributes much of total chromatin content [43].

To minimize non-specific ChIP signal resulting from a single antibody, we used three different anti-Pol II C-terminal domain (CTD) YSPTSPS peptide antibodies, each corresponding to a different phosphorylation state of the peptide: unphosphorylated [8WG16], phosphorylated at the Ser2 residue [S2], and phosphorylated at the Ser5 residue [S5]. For this analysis, we constructed and sequenced a total of six Pol II ChIP-seq libraries, as well as two ChIP input libraries using the Illumina platform. An average of 2.1 million reads that perfectly aligned to the *C. elegans* genome were obtained (approximately 4× coverage).

**Figure 1:**
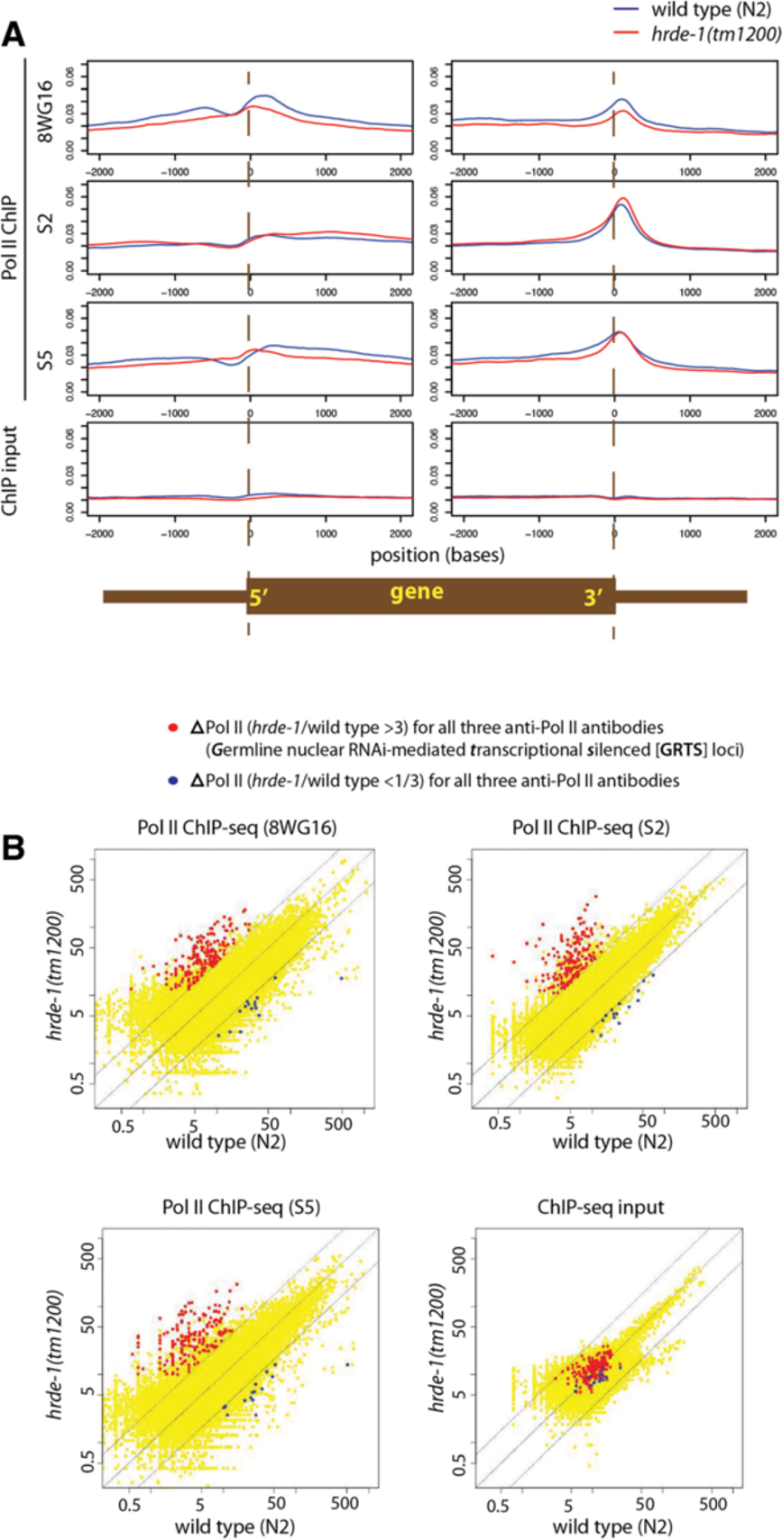
**Loss of HRDE-1 increases RNA polymerase II (Pol II) occupancy at specific genomic loci. (A)** Comparison of averaged Pol II occupancy levels in the wild type and *hrde-1(tm1200)* mutant animals around the 5′ and 3′ ends of 3903 “H3K4me2/3-anchored” genes [44]. Left panels: Pol II ChIP or ChIP input signal was plotted as a function of distance to the dyad of the “plus-one” nucleosome, defined by peak H3K4me2/3 nucleosomes near transcription start sites for the 3903 ‘H3K4me2/3-anchored’ genes. Right panels: Pol II ChIP or ChIP input signal was plotted as a function of distance to the annotated ends of 3′ UTR. **(B)** Pol II ChIP or ChIP input signals for the wild type and *hrde-1(tm1200)* mutant animals on 100,257 1-kb regions in the *C. elegans* genome. Each dot corresponds to a 1-kb region. Dotted lines indicate no-changes (the middle line) and three-fold differences (the flanking lines).

Our assay was validated by the strong relative enrichment of Pol II ChIP signals at both the 5′ and 3′ ends of a set of “H3K4 di- or tri-methylation-anchored” genes, which represent actively transcribed genes in *C. elegans* [44] (Figure 1A). In each of the three Pol II ChIP-seq experiments (8WG16, S2, and S5), the averaged Pol II occupancy for the actively transcribed genes in the *hrde-1* mutant showed only modest differences from the one in the wild type (modest decrease for 8WG16 and S5 and modest increase for S2 in the *hrde-1* mutant). The differences were even smaller in a similar metagene analysis for all annotated protein-coding genes (data not shown).

To expand our analysis at a higher resolution for the entire genome, we divided the genome into 100,257 1-kb segments and determined the Pol II ChIP-seq signal for each segment. Sequenced reads that were aligned to repetitive regions in the genome were normalized by the frequency that a non-unique read aligns to a different position in the genome. For most of the genome, the wild type and *hrde-1* mutant exhibited similar levels of Pol II occupancy (Figure 1B). By using a 3-fold cutoff, the *hrde-1* mutant animals showed consistent increases in Pol II occupancy at 191 1-kb regions in all three sets of Pol II ChIP samples (8WG16, S2, and S5) (Figure 1B and Additional file 1: Table S1). The median fold of increase in Pol II occupancy for these loci was 5.3 (8WG16), 5.8 (S2), and 9.0 (S5), indicating HRDE-1′s prominent role in transcriptional silencing for at least 0.2% of the *C. elegans* genome. We provisionally refer to these 191 1-kb regions as the exemplary *g*ermline nuclear *R*NAi-dependent *t*ranscriptional *s*ilencing (GRTS) loci. Regions with a minimal 2-fold and 1.5-fold increase in the Pol II ChIP signals (*hrde-1* vs. wild type) were listed in Additional file 1: Table S1. We found a much smaller number of 1-kb regions (15) which showed decreased levels of Pol II ChIP-seq signals in the *hrde-1* mutant animals. Most of these changes were modest (Figure 1B) and most likely corresponded to experimental noise or indirect effects of the *hrde-1* mutation. They were not further analyzed in this work.

**Figure 2:**
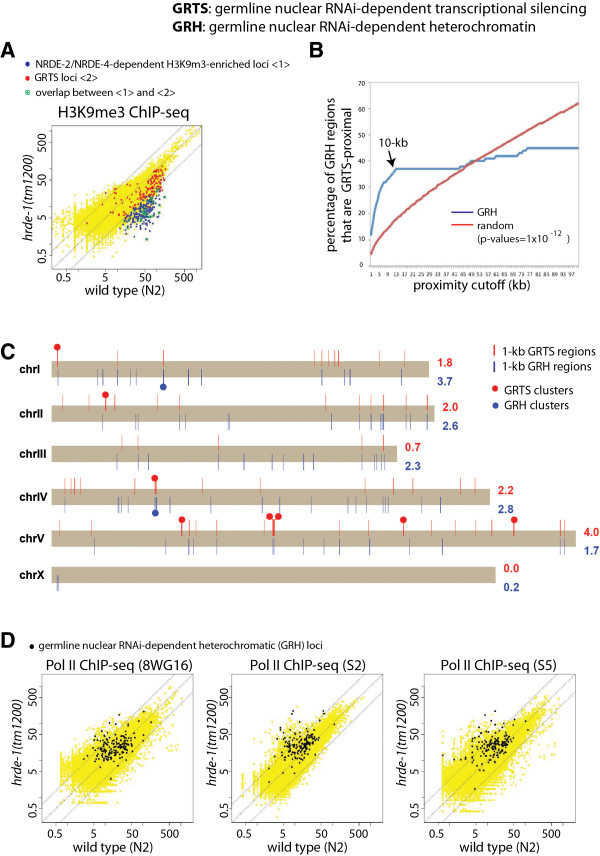
**Germline nuclear RNAi-dependent heterochromatic (GRH) regions. (A)** H3K9me3 ChIP-seq signals for the wild type (N2) and *hrde-1(tm1200)* mutant on 100,257 1-kb regions in the *C. elegans* genome. GRH and GRTS loci were indicated by blue and red dots, respectively. **(B)** Blue curve: the percentage of the 215 1-kb GRH regions that are nearby to or overlap with any GRTS as a function of the proximity cutoff. Red curve: the minimal percentage to reject the null hypothesis that the 215 1-kb GRH regions have no tendency to overlap with or nearby to GRTS regions (p-values = 1 × 10^−12^, binomial distribution) as a function of the proximity cutoff. **(C)** Genomic distribution of GRTS (red bars) and GRH (blue bars) regions in each of the six chromosomes in *C. elegans*, with prominent GRTS clusters and GRH clusters (at least 10 kb) indicated with solid circles. Numbers on the right of each chromosome are the percentages of the chromosome in GRTS (red) and GRH (blue) regions. **(D)** Scatter plots of the whole-genome Pol II ChIP-seq signals for the wild type and *hrde-1(tm1200)* mutant animals with the GRH regions highlighted (otherwise the same data as used in Figure 1B). Dotted lines **(A and** **D** **)** indicate no-changes (the middle line) and three-fold differences (the flanking lines).

The genomic distribution of the GRTS loci is not random; many GRTS loci are in proximity to each other. By setting a proximity cutoff of 5 kb, we found that 161 of the 191 exemplary GRTS loci (84.2%) are clustered in 35 different regions, here after referred to as GRTS clusters (Figure 2C). Eight of these 35 clusters were at least 10 kb, a size three times larger than the median size of *C. elegans* genes. In addition, all of the exemplary GRTS loci are located in five autosomes and strikingly absent in the sex chromosome (X) (Figure 2C). *C. elegans* has two sexes: hermaphrodite (XX) and male (X). Previous studies have shown that the entire X chromosome, with the exception of the left tip, is in a repressive chromatin state in *C. elegans* germ cells [45, 46]. Therefore, the depletion of GRTS loci in the X chromosome may reflect a paradox in the nuclear RNAi field [24], which states that the target locus needs to be at least transiently transcribed in order to be targeted for transcriptional silencing. Alternatively, the X chromosome in *C. elegans* germ cells may lack certain DNA sequences or chromatin components that are necessary for nuclear RNAi.

### Germline nuclear RNAi-dependent heterochromatin (GRH) loci and transcriptional silencing (GRTS) loci are in proximity to each other

We then asked to what extent the germline nuclear RNAi-dependent transcriptional silencing events are correlated with the heterochromatic responses on the native targets. In a previous study, we identified a set of nuclear RNAi-dependent heterochromatic regions (marked by H3K9 trimethylation) that are dependent on two different nuclear RNAi protein factors NRDE-2 and NRDE-4 [38]. To augment the previous study, we performed the H3K9me3 ChIP-seq analysis using the *hrde-1(tm1200*) mutant adult animals in this study. We found that the *hrde-1* mutant had very similar H3K9me3 defects to the *nrde-2* and *nrde-4* mutants. Using a 3-fold cutoff, we found that 215 out of the 358 NRDE-2/NRDE-4-dependent H3K9me3-enriched regions (60%) were dependent on the HRDE-1 activity as well. We refer to these 215 1-kb regions as exemplary *g*ermline nuclear *R*NAi-dependent *heterochromatic* (GRH) loci (Figure 2A) (listed in Additional file 2: Table S2). Similarly to GRTS (listed in Additional file 3: Table S3), nearly all of the GRH regions are located in the five autosomes and only three 1-kb GRH regions (1.4%) were located in the X chromosome (Figure 2C).

We compared the genomic distributions of the exemplary 215 GRH and 191 GRTS 1-kb loci and found that 35% of the GRH loci were within 10 kb distance of a GRTS locus (Figure 2B and 2C, p-values <1×10^−12^). Furthermore, approximately 50% of these GRH loci either overlapped with or were adjacent to a GRTS locus. These overlapping or proximally located GRTS and GRH loci represent a set of native targets at which germline nuclear RNAi triggers robust responses in both transcriptional silencing and H3K9 trimethylation.

Intriguingly, many of the GRTS loci, at which the *hrde-1* mutation leads to a dramatic transcriptional desilencing, had fewer H3K9me3 defects than the GRH loci (Figure 2A). In the *hrde-1* mutant, the median fold-of-reduction of H3K9me3 for GRH was 6.1, while the reduction for GRTS was only 2.5. Conversely, the *hrde-1* mutation had a much weaker effect on Pol II exclusion in the GRH loci than the GRTS loci. The median folds-of-increase of Pol II for GRH loci were 1.3, 1.9, and 2.0 for 8WG16, S2, and S5, respectively. In contrast, the increases for GRTS loci were 5.3, 5.8, and 9.0 for 8WG16, S2, and S5, respectively. In addition, many GRH loci exhibited no changes in Pol II occupancy between the wild type and *hrde-1* mutant animals (Figure 2D). These results indicate that the responses of germline nuclear RNAi may differ among the native targets. Furthermore, these results ruled out a simple model in which the level of H3K9 methylation is the sole determinant of transcription silencing at the native targets of germline nuclear RNAi.

### Impact of germline nuclear RNAi on the primary transcriptome

To further characterize the transcription activity, particularly its directionality, we decided to characterize pre-mRNA at the whole genome level. Previously, a nascent transcript sequencing method was developed to globally analyze pre-mRNA in *Saccharomyces cerevisiae* [47]. In this method, pre-mRNA was enriched by Pol II immunoprecipitation (IP) from DNase I-treated nuclear extract without crosslinking. We applied this method for *C. elegans* and found that >99% of Pol II remained insoluble even after extensive DNase I treatment of the crude nuclear extract (data not shown). We subsequently learned from previous works that, in metazoans, the ternary complex of RNA polymerase II, DNA, and nascent RNA is resistant to high concentrations of detergents, chaotropes, salt, and polyanions [48, 49]. So we modified the standard Pol II (S2) ChIP procedure and extracted RNA from the final IP product (see Methods for details), followed by RNA-seq analysis. We will refer to this method as pre-mRNA-seq in this work.

**Figure 3:**
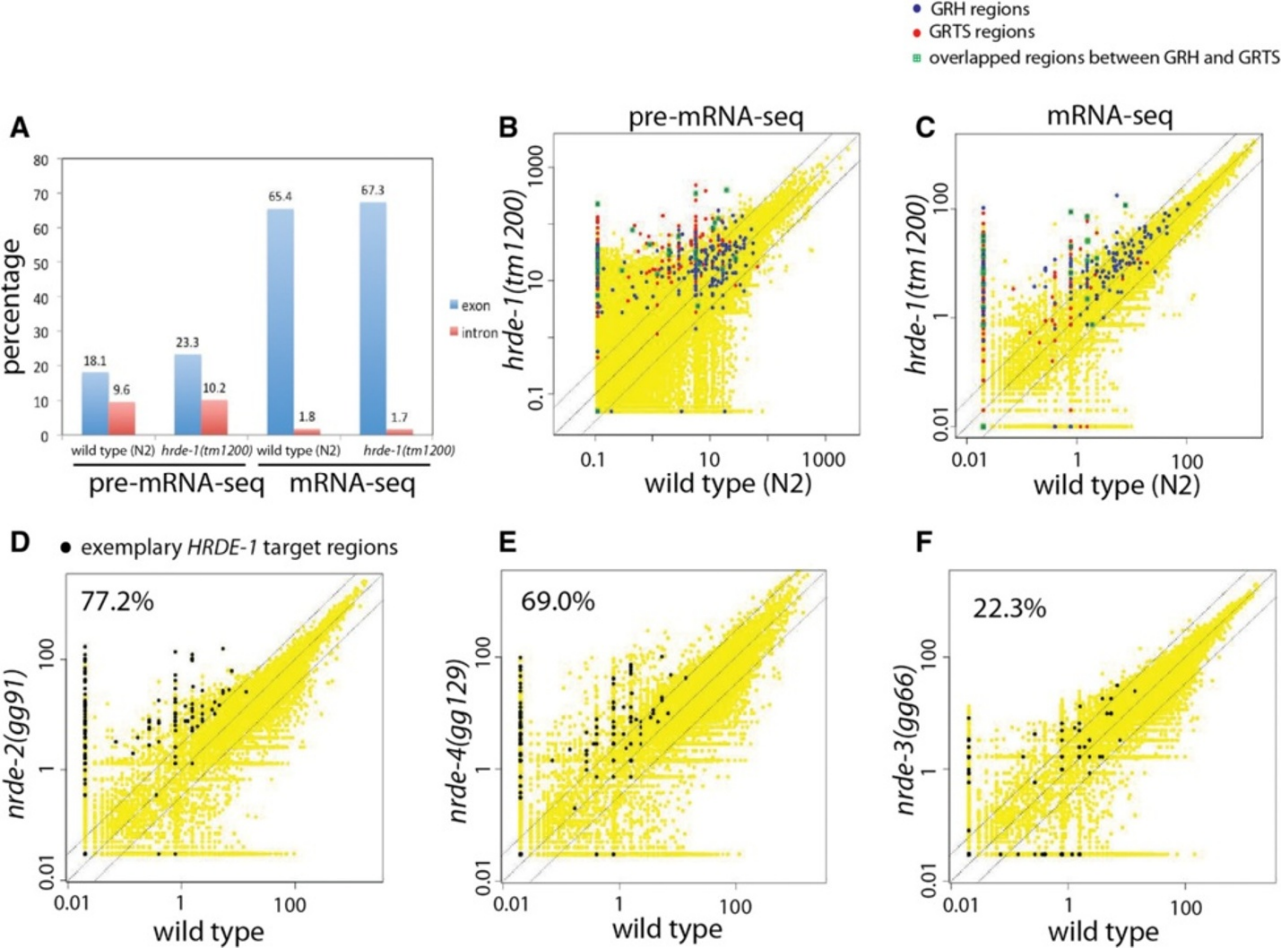
**Loss of HRDE-1 increases the levels of pre-mRNA and mRNA transcribed from the GRTS regions. (A)** Percentages of sequenced tags (pre-mRNA-seq or mRNA-seq) that match to sequences of exons or introns. Scatter plots of the pre-mRNA-seq **(B)** or mRNA-seq signals **(C)** for the wild type and *hrde-1(tm1200)* mutant animals. **(D-F)** Scatter plots of the mRNA-seq signals for the wild type, *nrde-2*(*gg91*)*, nrde-3*(*gg66*) *and nrde-4*(*gg129*) mutant animals. The percentage of the exemplary HRDE-1 targets showed at least 3-fold increases in mRNA-seq signals in each mutant was indicated in each panel.

**Figure 4:**
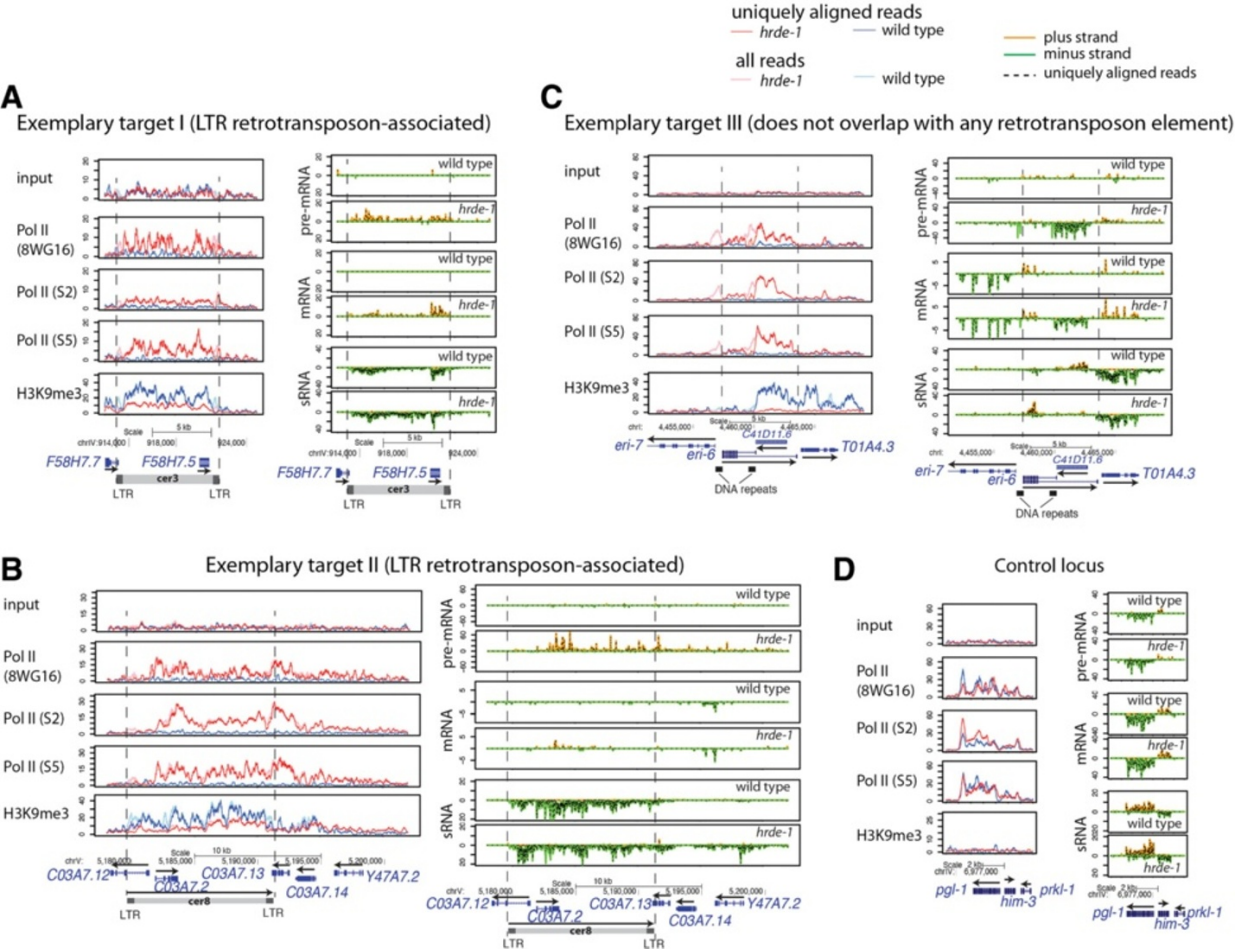
**Exemplary regions with HRDE-1-dependent transcriptional silencing and heterochromatin responses.** Pol II ChIP-seq, H3K9me3 ChIP-seq, pre-mRNA-seq, mRNA-seq, and small RNA-seq profiles are plotted for three germline nuclear RNAi targets (*Cer3* **(A)**, *Cer8* **(B)** and *C41D11.6* **(C)**) and one control locus **(D)**.

To verify that pre-mRNA, not mRNA, was indeed enriched by this method, we searched for intronic sequences in the pre-mRNA-seq reads. We found that intronic reads were largely increased in the pre-mRNA-seq samples when compared with the matching mRNA-seq samples (Figure 3A). The [intron/exon] ratio of the pre-mRNA-seq was 19.3-fold higher than the ratio of the mRNA-seq for the wild type and 17.6-fold higher for the *hrde-1* mutant. This verifies an efficient enrichment of primary transcripts by the pre-mRNA-seq method. We also observed regions with abundant pre-mRNA-seq reads but very low mRNA-seq reads (*e.g.,* Figure 4B, comparing pre-mRNA and mRNA for the *hrde-1* mutant sample), evidencing a unique power of pre-mRNA-seq in revealing gene expression activity. In addition, pre-mRNA-seq compliments the Pol II ChIP-seq analysis by indicating the directionality of transcription activity.

We examined the pre-mRNA-seq signals from the wild type and *hrde-1* mutant animals for all 1-kb segments throughout the genome (Figure 3B). Among the 191 exemplary GRTS loci, 61.8%, 35.1%, and 17.3% showed at least 10, 50, and 200-fold increase in pre-mRNA-seq signals in the *hrde-1* mutant over the wild type animals (p-values < 0.0005, Monte Carlo simulation), evidencing a large increase in nascent transcripts in the GRTS regions. Consistent with Pol II ChIP-seq analysis, pre-mRNA-seq analysis revealed a generally weaker transcription de-silencing response in the GRH regions than in the GRTS regions. Among the 164 GRH loci, 25.6%, 10.9%, and 5.1% of them showed 10, 50, and 200-fold increases, respectively, in pre-mRNA-seq signals in the *hrde-1* mutant compared to the wild type animals (p-values: 0.92, 0.72, and 0.002, Monte Carlo simulation). A weaker desilencing in the *hrde-1* mutant in GRH regions was also evident from the mRNA-seq analysis (Figure 3C).

To test whether HRDE-1-dependent silencing events also require other nuclear RNAi pathway genes, we performed mRNA-seq analysis for mutants of three other core nuclear RNAi factors [*nrde-2*(*gg91*)*, nrde-3*(*gg66*) *and nrde-4*(*gg129*)]*.* For the mRNA-seq analysis, we defined a set of exemplary HRDE-1 targets as the combined GRTS and GRH regions with increased mRNA-seq signals in *hrde-1* mutant animals (a minimal of 3-fold increase in the *hrde-1* mutant compared with the wild type mRNA-seq samples). We found that both the *nrde-2* mutant and the *nrde-4* mutant showed strong de-silencing of the exemplary HRDE-1 target regions: 77.2% and 69.0% of the exemplary HRDE-1 targets showed at least 3-fold increases in mRNA-seq signals in the *nrde-2* mutant and *nrde-4* mutant, respectively, when compared with the wild type (Figure 3D and E). By contrast, only 22.3% of the exemplary HRDE-1 targets showed at least 3-fold increases in mRNA-seq signals in the *nrde-3* mutant (Figure 3F). We previously found that the soma-specific nuclear Argonaute protein NRDE-3 is not required for the endogenous H3K9me3 responses mediated by NRDE-2 and NRDE-4 [38]. Taken together, these results further confirm that NRDE-3 has little or no function in germline nuclear RNAi.

### Germline nuclear RNAi primarily targets retrotransposons for transcriptional silencing

To gain insight into the physiological role of germline nuclear RNAi in *C. elegans*, we examined the top sixteen largest GRTS clusters (5–19 kb) for their genomic annotations. Although protein-coding genes in these clusters were not associated with any obvious common feature, we found that LTR retrotransposons were highly enriched in the GRTS clusters. 10 of these 16 GRTS clusters contain either full or partial LTR retrotransposon-type elements (p-value = 0.0005, Monte Carlo simulation) (Table 1). This strong association was further evidenced by a close correspondence between the locations of target LTR retrotransposons and the local profiles of endo-siRNAs in both the wild type and *hrde-1* mutant (see next section), H3K9me3 in the wild type, and Pol II occupancy/pre-mRNA in the *hrde-1* mutant (two examples were shown in Figure 4A and 4B). We note that the top 16 largest GRTS clusters were used for this analysis because (1) full-length LTR retrotransposons in *C. elegans* are usually larger than 8 kb and (2) these larger clusters represent a high-confidence reference data set of HRDE-1 targets. Some of the smaller GRTS regions also contain retrotransposon fragments (data not shown). Within each of these LTR retrotransposons, nearly all of the pre-mRNA reads in the *hrde-1* mutant sample were mapped to the sense strand of the protein-coding genes within the targets (*e.g.,* Figure 4A and 4B). Together with profiles of Pol II-ChIP-seq and pre-mRNA-seq in the *hrde-1* mutant, the strong transcription directionality strongly suggests that transcription of these LTR retrotransposons initiates from the upstream LTR (relative to the sense strand of protein-coding sequence within the transposons) and terminates within or near the downstream LTR.

**Table 1:** **Top sixteen largest germline nuclear RNAi-dependent transcriptional silencing (GRTS) clusters**

Genomic position (WS190)	Length (kb)	LTR retrotransposon	LINE	Tc/mariner-type DNA transposon	Other types of DNA repeat	Protein-coding genes	GRH
chrI:226000-239000	13	A *Cer13* fragment (656 bp) with homology to the PAO family retrotransposon integrase. No LTR at this target.	-	-		*Y48G1BM.5, Y48G1BM.6, Y48G1BM.7*	+
chrI:4456000-4464000	8	-	-	-	Two ~930 bp DNA repeats that are 1.9 kb apart and 99% identical (chrI:4456382–4457314 and chrI:4459215–4460136). One of the two repeats is located between *eri-6* and *eri-7* and contains the promoter active for the two genes.	*eri-7, eri-6, C41D11.6, T01A4.3*	+
chrI:11438000-11449000	11	*Cer16-2* (9.3 kb) with LTR in both ends	-	-		*C27C7.1*	+
chrII:2150000-2163000	13	*Cer9-1* (9.5 kb) with LTR in both ends	-	-		*C40A11.8, math-18*	+
chrII:2516000-2521000	5	-	-	-	Two ~430 bp DNA repeats that are 3.5 kb apart and 98% identical to each other (chrII:2,520,235-2,520,670 and chrII:2516272–2516702)	*bath-12, bath-13*	+
chrII:13245000-13250000	5	-	-	-		*F15D4.5, F15D4.6*	+
chrIV:915000-922000	7	An extensive *Cer3-1* (8.7 kb) with LTR in both ends	-	-		*F58H7.5, F58H7.6*	+
chrIV:4124000-4138000	14	A solo LTR (*Cer2*) with four other *Cer2* fragments (275 bp-412 bp)	-	-		*F49F1.7, F49F1.8*	+
chrIV:13633000-13639000	6	-	LINE2C1 fragment	-		*C08F11.5, C08F11.7*	+
chrV:5182000-5201000	19	*Cer9-1* (11.5 kb) with LTR in both ends	-	-		*C03A7.12, C03A7.2, C03A7.13, abu-8, Y47A7.2*	+
chrV:5482000-5487000	5	-	-	-		*srg-66, srg-65, grl-18*	+
chrV:8826000-8837000	11	Mixed *Cer8* and *Cer9* sequence (19.8 kb) with LTR (cer9) in both ends,	-	-		*F09B7.3, W09B7.2 (F07B7.2), W09B7.1 (F07B7.1), F07B7.7, F07B7.8*	+
chrV:8868000–8879000 (identical sequence to chrV:8826000–8837000)	11	Mixed *Cer8* and *Cer9* sequence (19.8 kb) with LTR (cer9) in both ends,	-	-		*F09B7.3, W09B7.2 (F07B7.2), W09B7.1 (F07B7.1), F07B7.7, F07B7.8*	+
chrV:14039000-14055000	16	-	LINE2H	-		*T08G5.9, T08G5.8, T08G5.7, F58D12.5, T08G5.19*	-
chrV:17568000-17573000	5	A *Cer8-1* fragment (68 bp)	-	-		*C38D9.2*	+
chrV:18439000-18457000	18	*Cer8-1* (19.4 kb) with LTR in both ends	-	*Tc1* embedded in the *Cer8-1*		*ZK262.8, ZK262.9, ZK228.1, ZK228.10*	+

In contrast to mammals and plants, retrotransposons in the *C. elegans* are sparse and occupy only approximately 0.4% of the genome [50–52]. Furthermore, retrotransposition in *C. elegans* has never been reported [52]. However, DNA transposons, which are active in transposition in *C. elegans*, occupy a much larger fraction (approximately 10%) of the *C. elegans* genome [52]. We found that there was only one GRTS cluster that had a DNA transposon (Tc1), which is embedded in an LTR retrotransposon in this case (Table 1). These results indicate that LTR retrotransposons, but not DNA transposons, are a major class of germline nuclear RNAi targets.

### The native targets of germline nuclear RNAi are associated with abundant endo-siRNAs

**Figure 5:**
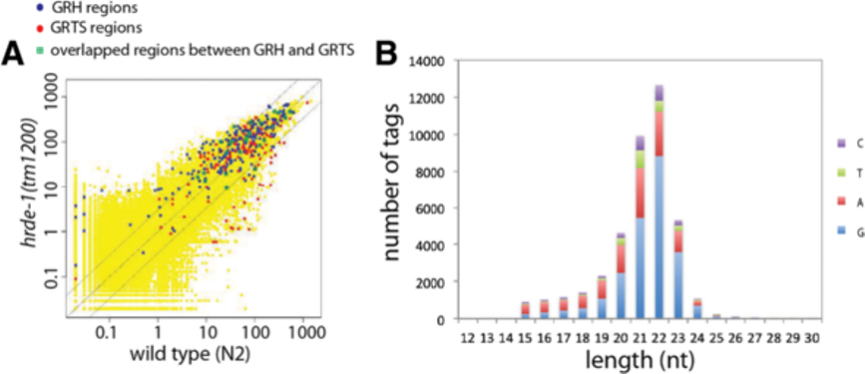
**HRDE-1 is not required for the biogenesis of germline nuclear RNAi-associated endo-siRNA. (A)** Scatter plots of the small RNA-seq signals for the wild type and *hrde-1(tm1200)* mutant animals. Each dot represents each of the 100,257 1-kb regions, with GRTS, GRH, and overlapped regions between GRTS and GRH highlighted. Dotted lines indicate no-changes (the middle line) and three-fold differences (the flanking lines). **(B)** The size distribution and nucleotide distribution for the first position at the 5′ end of small RNAs in the GRTS and GRH regions.

To examine endo-siRNAs associated with native targets of germline nuclear RNAi, we performed small RNA sequencing analyses using wild-type adult animals and *hrde-1* mutant adults. We used a 5′-mono-phosphate (mono-Pi)-independent method to capture small RNAs with mono-Pi or tri-Pi at their 5′ ends. As expected for RNAi-regulated loci, we found that the native target regions of germline nuclear RNAi were associated with abundant endo-siRNAs (Figures 4A-C and 5A). Small RNAs that were mapped to these regions measured predominantly 21 or 22 nucleotides long, began with a G at the 5′ end (Figure 5B), and were antisense to the primary transcripts (Figure 4A-C). These features indicate that these endo-siRNAs belong to the so-called 22G RNA family and are products of RdRPs [9, 10, 53].

We also compared the endo-siRNA profiles between the wild type and *hrde-1* mutants. The overall abundance of GRTS- or GRH-corresponding endo-siRNAs were similar between these two samples (Figure 5A). This result indicates that HRDE-1 does not directly function in the endo-siRNA biogenesis pathway. Nevertheless, some differences were observed between the wild type and the *hrde-1* mutant when endo-siRNA profiles were examined in detail along the target sites. Possibly these differences correspond to a secondary effect of transcriptional de-silencing caused by the *hrde-1* mutation.

### Non-retrotransposon GRTS regions

In addition to LTR retrotransposons, we also identified several GRTS clusters that do not contain any annotated retrotransposons. One prominent example covers the *C41D11.6* gene (Figure 4C), which exhibits strong increases in Pol II occupancy, pre-mRNA levels, and mRNA levels in the *hrde-1* mutant animals. *C41D11.6* also exhibits a high degree of HRDE-1-dependent H3K9 trimethylation, which extends into a neighboring, divergently transcribed gene (*T01A4.3*). These two genes appear to be nematode-specific genes with unknown functions.

On the other side of C41D11.6 is a locus encoding the *eri-6* and *eri-7* genes (Figure 4C). Despite the divergent transcription of *eri-6/eri-7*, their mRNAs are joined together by an unusual trans-splicing event [54]. Intriguingly, by performing qRT-PCR using primers specific to the *eri-6/7* trans-spliced product, we observed a 50% reduction in the *eri-6/7* mRNA level in the *hrde-1* mutant, suggesting the existence of a local effect of germline nuclear RNAi on the expression of a neighboring gene. We found that loss of eri-6/7 had no silencing defects on the natural targets of germline nuclear RNAi [data not shown].

### The piRNA pathway is not required for germline nuclear RNAi responses at the native targets

The piRNA pathway in *C. elegans* plays an important role in silencing certain pseudogenes, DNA transposons, and other types of foreign DNA such as transgenes in the germline [55–59]. The broad targeting capability comes from the highly diverse repertoire of sequences encoded by piRNAs (also called 21U RNAs) in *C. elegans*. piRNA-mediated silencing of transgenes was previously shown to be dependent on HRDE-1 [40, 41].

**Figure 6:**
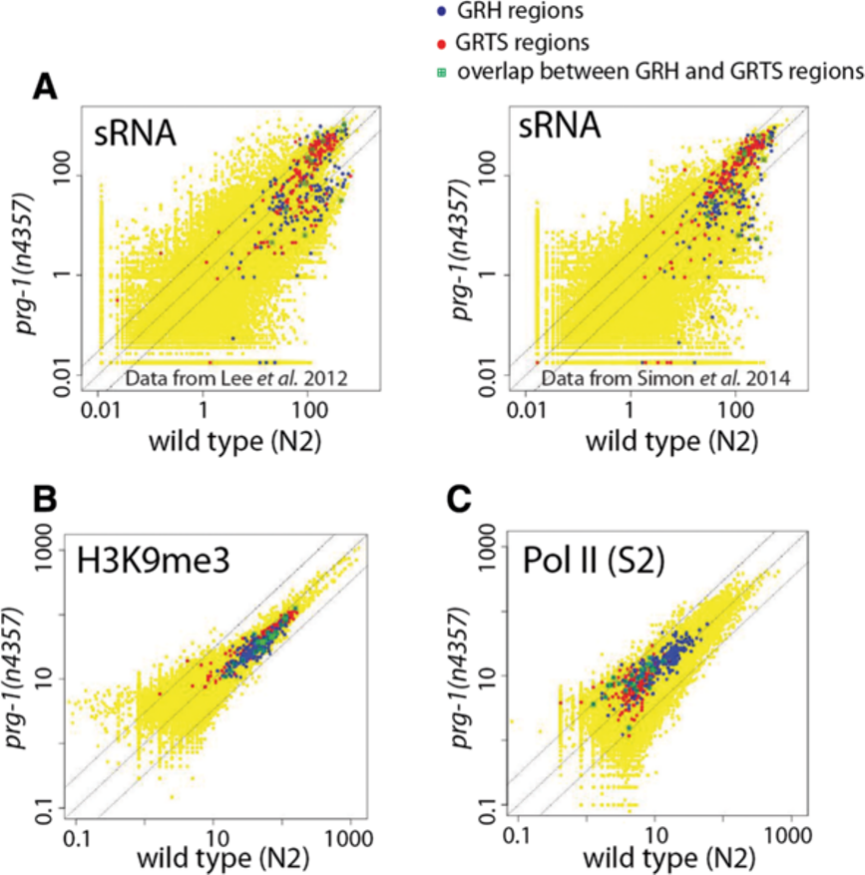
**The piRNA pathway is not required for germline nuclear RNAi responses at the native targets.** Scatter plots of small RNA-seq signals **(A)**, H3K9me3 ChIP-seq signals **(B)**, and Pol II (S2) ChIP-seq signals **(C)** for the wild type and *prg-1*(*n4357*) mutant animals. Each dot represents each of the 100,257 1-kb regions, with GRTS, GRH, and overlapped regions between GRTS and GRH highlighted. Dotted lines indicate no-changes (the middle line) and three-fold differences (the flanking lines).

To investigate whether the piRNA pathway is required for germline nuclear RNAi activity on the native targets, we performed genome-wide analyses using a piRNA pathway mutant. The *C. elegans* genome encodes two PIWI family proteins, PRG-1 and PRG-2*.* Previous studies found that PRG-1 is required for the stability of piRNAs and essential for piRNA activity. By contrast, PRG-2 has very little or no function in the piRNA pathway [11, 56, 60]. Previous studies found that a subset of the endogenous 22G RNA population is dependent on the piRNA pathway [57, 58]. To ask whether regions with PRG-1-dependent endo-siRNAs overlap with the exemplary GRH and GRTS regions, we examined two sets of published small RNA-seq data for the *prg-1*(*n4357*) mutant and matching wild type animals [58, 61]. By using a 3-fold cutoff, we found that only 7.5% and 20.6% of the exemplary GRTS and GRH regions, respectively, had reduced levels of endo-siRNAs in the *prg-1* mutants. The reduction for most of these regions was modest (Figure 6A). These results indicate that endo-siRNA levels for the majority of the natural targets of germline nuclear RNAi are not dependent on the piRNA pathway.

We then asked whether loss of *prg-1* had any effect on the germline nuclear RNAi-mediated heterochromatin response and transcriptional silencing at the native target sites. For this we performed H3K9me3 and Pol II (S2) ChIP-seq experiments using the *prg-1(n4357)* mutant animals. The results showed that loss of PRG-1 had virtually no effect on the H3K9me3 or Pol II occupancy profiles on the native targets of germline nuclear RNAi (Figure 6B and C), indicating that germline nuclear RNAi in *C. elegans* does not require the piRNA pathway.

## Conclusions

### Retrotransposon silencing

By taking a combined genetic, biochemical, and computational approach, we identified a distinct set of genomic regions that are naturally targeted by germline nuclear RNAi for transcriptional silencing and/or heterochromatin formation in *C. elegans*. The enrichment of retrotransposon elements in these regions suggests that germline nuclear RNAi evolved as a genome defense mechanism against this type of mobile DNA. Our detailed whole-genome profiling of Pol II occupancy and pre-mRNA indicates that, first, the HRDE-1-targeted retrotransposons in *C. elegans* are intrinsically active in transcription. Second, germline nuclear RNAi-mediated transcriptional silencing at these regions occurs prior to (or at) the formation of the transcriptional preinitiation complex. Taken together, these findings indicate that one function of germline nuclear RNAi is to silence the retrotransposons in the *C. elegans* genome.

### piRNA

Studies using *Drosophila*, zebrafish, and mice have revealed that the piRNA pathway plays a central role in silencing retrotransposons at both the transcriptional and post-transcriptional levels. piRNAs in *C. elegans* (also called 21U RNA) have been shown to silence Tc3 (a mariner-type DNA transposon), certain pseudogenes, and transgenes in the germline [56–58]. Previous studies indicated that HRDE-1 is required for the silencing of piRNA-targeted reporter genes [40, 41]. In this study, we showed that the piRNA pathway in *C. elegans* is not required for germline nuclear RNAi activity at LTR retrotransposons. These findings suggest that the HRDE-1-mediated nuclear RNAi and the piRNA pathway evolved to have distinct target specificity in genome surveillance.

### Triggers of nuclear RNAi

The triggering mechanisms that initiate RNAi-mediated transcriptional silencing appear to be quite diverse in different organisms. In plants, nuclear RNAi can be triggered *in trans* by dsRNA produced from an infecting virus or transgene [62, 63]. Cryptic splicing, aberrant transcription products, and transcription-blocking activities (such as DNA replication) have been shown to play important roles in triggering RNAi-mediated chromatin silencing *in cis* in fission yeast [64–66].

The native targets of germline nuclear RNAi in *C. elegans* identified in this study often contain DNA repeats that are associated with promoter activities (*e.g.,* LTRs). It is possible that certain unusual nucleic acid interactions (RNA-RNA, RNA-DNA, or DNA-DNA) associated with transcriptionally active DNA repeats are recognized by the host as signals for unwanted genetic elements. In the case of the *eri-6/eri-7* locus, although no DNA transposon or retrotransposon can be found nearby, there are several unusual features of gene structures: (1) an approximately 0.9 kb DNA sequence that includes the promoters for *eri-6* and *eri-7* is duplicated 3 kb away in the same orientation (Figure 4C), which resembles of LTRs; (2) the partially overlapped genes *C41D11.6* and *eri-6* are transcribed in convergent directions; (3) and the C*41D11.6* gene is an intron-less gene. We are currently investigating whether nuclear RNAi at this region is caused by any of these features.

A previous study found that at least one of the LTR retrotransposons, namely *Cer1*, is actively transcribed in germ cells of wild type *C. elegans* [67]. We observed the active expression of *Cer1* in the wild type in our data set as well (data not shown). This suggests that DNA repeats, such as LTR, may not be sufficient to trigger silencing. Other genetic activities or molecular structures that are intrinsic to retrotransposons may be required as well. Alternatively, the triggering mechanism may involve interference between genetic activities (*e.g.,* transcription and DNA replication) that originates from foreign DNA and neighboring host genes. Such interference may be absent in the *Cer1* locus. We consider these models to be valid hypotheses for further study.

### Absence of response in the X chromosome

With a few exceptions, GRTS and GRH regions are situated in all five autosomes but not on the X chromosome. What are the possibly explanations for their absence in the X chromosome? Our previous study showed that abundant small RNAs from both types of chromosomes are associated with HRDE-1 [38]. Therefore, this positioning cannot be due to the lack of X-chromosome endo-siRNAs. LTR retrotransposons themselves are not absent in the X chromosome either. Some of the X-located LTR elements are nearly identical to ones located in the GRTS regions. At this point we cannot rule out the lack of certain intrinsic triggering sequences in the X-located LTR retrotransposons. It is also possible that the lack of X-located GRTS or GRH regions is due to the global silencing of the X chromosome in the germ cells of *C. elegans.* Previous studies found that the entire X chromosome in *C. elegans* germ cells, with the exception of one tip of the X chromosome, is in a repressive chromatin state [45, 46]. It is conceivable that a brief period of transcription is needed to provide nascent transcripts as binding sites for targeting siRNA-associated silencing complex. Transient transcription may even be required to generate the triggering signals, for example, by providing nascent transcripts recognized as improperly spliced or terminated transcripts, or providing nascent transcripts as templates or substrates for siRNA biogenesis.

## Methods

### Worm strains

*C. elegans* strain N2 was used as the standard wild-type strain. Mutant alleles used in this study were *hrde-1*(*tm1200)* [38, 40, 41] *, nrde-2*(*gg91*) [36] *, nrde-3*(*gg66*) [35] *, nrde-4(gg129*) [37] and *prg-1*(*n4357*) [56]. Synchronized animals were cultured on NGM plates at 19°C and fed on *E. coli* OP50 [68].

Chromatin Immunoprecipitation (ChIP)-seq, pre-mRNA-seq, mRNA-seq, and small RNA-seq procedures are described in Additional file 4.

## Availability of supporting data

All sequencing data used in this study have been deposited in GEO (Accession Number: GSE58031).

## Electronic supplementary material

Additional file 1: Table S1: GRTS and GRH regions identified with cutoffs of 1.5, 2, or 3-fold differences. (XLSX 79 KB)

Additional file 2: Table S2: GRH clusters identified using data from Additional file 1: Table S1. Each of these regions has at least one 1-kb region with a minimal 3-fold decrease in H3K9me3 in all three nuclear RNAi mutants (*nrde-2, nrde-4*, and *hrde-1*) and also includes nearby regions with at least 1.5-fold decreases. Each cluster was indicated whether it overlaps with any GRTS cluster. Genes in each GRH cluster were listed. (XLSX 53 KB)

Additional file 3: Table S3: GRTS clusters identified using data from Additional file 1: Table S1. Each of these regions has at least one 1-kb region with a minimal 3-fold increase in Pol II ChIP-seq signal in *hrde-1* and also includes nearby regions with at least 1.5-fold increases. Each cluster was indicated whether it overlaps with any GRH cluster. Genes in each GRTS cluster were listed. (XLSX 53 KB)

Additional file 4: **Supplementary Methods.** (DOCX 52 KB)

## Abbreviations

GRTS: Germline nuclear *R*NAi-dependent *t*ranscriptional *s*ilencing
GRH: Germline nuclear *R*NAi-dependent *h*eterochromatin.
