# Supplementary material for: Complex coding of endogenous siRNA, transcriptional silencing and H3K9 methylation on native targets of germline nuclear RNAi in C. elegans
Source: BMC Genomics. 2014 Dec 22;15(1):1157. doi: 10.1186/1471-2164-15-1157 (PMC4367959; doi:10.1186/1471-2164-15-1157)
Supplement: Supplementary file 4 — Additional file 4: Supplementary Methods. (DOCX 52 KB) [file 12864_2014_6890_MOESM4_ESM.docx]

Supplemental information for “Complex coding of endogenous siRNA, transcriptional silencing and H3K9 methylation on native targets of germline nuclear RNAi in *C. elegans*”

Julie Zhouli Ni ^1^, Esteban Chen ^1^, and Sam Guoping Gu ^1,2^

^1^ Department of Molecular Biology and Biochemistry, Rutgers the State University of New Jersey, Piscataway, New Jersey, 08854, United States of America

^2^ Corresponding author

Phone: (732)-445-0824, Fax: (732)-445-6186, Email: ggu@dls.rutgers.edu

**Methods**

**Chromatin Immunoprecipitation (ChIP)**

100-200 μl frozen synchronized young adult worm pellets were used for each chromatin immnuoprecipitation experiment. Crushed pellets (pulverized by grinding in liquid nitrogen with a mortar and pestle) were resuspended in 1ml of pre-chilled RIPA buffer (1X PBS, 1% NP-40, 0.5% sodium deoxycholate, 0.1% SDS, and 1X HALT combined protease and phosphatase inhibitor cocktail [ThermoScientific]). To crosslink, formaldehyde was added to the crude extract to a final concentration of 2%. The lysate was rotated at 4°C for 10 minutes. 0.1 ml of 1M Tris-HCl (pH 7.5) was added to quench formaldehyde. Lysate was spun at 6000 x g for 30 second and the supernatant was removed. The pellet was resuspended in 0.5ml of pre-chilled RIPA buffer. The lysate was transferred to a 1.5 ml TPX tube (Diagenode) and sonicated (Bioruptor [Diagenode], output level: high, interval: 0.5, three times of 8-minute sonication). 50 μl of the lysate was used to make IP input library. For each immumoprecipiation experiment, 2 μg of anti-H3K9me3 (ab8898, Abcam), anti-Pol II-8WG16 (ab817, Abcam), anti-Pol II-S2 (ab5095, Abcam), or anti-Pol II-S5 (ab5131, Abcam) antibody was added to 400 μl of the lysate (containing approximately 50 μg DNA). The IP mix was rotated overnight at 4°C. 50 μl of Protein A Dynabeads (Life Technology) was added and rotated for another 2 hours. The beads were then washed three times with 800 μl ice-cold LiCl washing buffer (100 mM Tris-HCl, pH7.5, 500 mM LiCl, 1% NP-40 and 1% sodim deoxycholate). To elute the immunoprecipitation product and reverse crosslink, beads were incubated with 400 μl of worm lysis buffer (0.1 M Tris-HCl, pH 7.5, 100 mM NaCl, 50 mM EDTA, 1% SDS, and 200 μg/ml of proteinase K) at 65 °C for 2 hours with agitation every 30 minutes (IP input lysate was treated similarly to reverse crosslink) and then subject to organic extraction and precipitation of DNA and RNA. DNA libraries were prepared as previously described [[1](#_ENREF_1)].

**Pre-mRNA-seq**

To enrich Pol II-associated nascent transcripts, Pol II ChIP was performed using anti-Pol II-S2 (ab5095, Abcam) antibody (see previous paragraph for ChIP procedure). DNA was removed from Pol II ChIP product by DNase I treatment according to manufacture’s instruction (New England Biolabs). RNA was then fragmented using Fragmentation Reagents (Ambion). To prepare pre-mRNA libraries, fragmented RNA was treated T4 polynucleotide kinase (New England Biolabs). The 3’ end of RNA was ligated to an adenylated DNA oligo (IDT linker-1: 5’-rAppCTGTAGGCACCATCAATC-3’) using T4 RNA ligase 1 (New England Biolabs) without ATP. ~15-35 nt RNA captured by the 3’ linker oligo was purified using 8 % polyacrylamide (acrylamide:bis-acrylamide = 19:1) gels containing 8 M urea, followed by a 5’ end ligation reaction using a DNA-RNA hybrid oligo (5’-ACGCTCTTCCGATCTrNrNrNrN-3’, rNrNrNrN as 4-nt barcode) using T4 RNA ligase 1 (New England Biolabs) with ATP. cDNA was synthesized by using SuperScript III Reverse transcriptase (Life Technologies) and primer SG460 (5’-GGAGTTCAGACGTGTGCTCTTCCGATCTATTGATGGTGCCTACAG-3’), followed by PCR amplification with primers SG-465 (5’-AATGATACGGCGACCACCGAGATCTACACTCTTTCCCTACACGACGCTCTTCCGATCT-3’) and SG-46X (5’-CAAGCAGAAGACGGCATACGAGAT[6-nt index]GTGACTGGAGTTCAGACGTGTGCTCTTCC-3’). To ensure that amplified DNA is still annealed to a true complement and avoid reannealing-distortion in the resulting libraries [[2](#_ENREF_2)], PCR reactions were titrated with increasing cycle numbers; cycle numbers for which product levels have not saturated (*i.e.* product levels still able to increase substantially with additional cycles) were selected. After separating PCR products on 3 % agarose gels, DNA bands of the expected size were extracted (QIAchange® Gel Extraction Kit [Invitrogen]; omitting the 50°C heating step), followed by massive parallel DNA sequencing (Illumina HiSeq2000).

**mRNA-seq**

Total RNA was extracted from frozen synchronized adult worm pellets (100-200 μl, crushed) using Trizol reagent (Life Technologies). mRNA was enriched using Poly(A)Purist MAG kit (Life Technologies). 0.5-1 μg of mRNA was used for each mRNA-seq library. The procedure of mRNA-seq library construction was the same as pre-mRNA-seq library construction described in this paper, except that 35-60 nt mRNA fragments were selected for library preparation.

**Small RNA-seq** was performed using a 5’-monophosphate-independent small RNA cloning procedure described in [[1](#_ENREF_1)].

**qRT-PCR**

Total RNA was isolated from 200~500 synchronized adult worms by Trizol reagent (Life Technologies) according to the manufacture’s instructions. To remove DNA, total RNA was incubated with 1U of DNase I (New England Biolabs) at 37°C for 30 minutes in 1X DNase I Reaction Buffer. DNase I was then inactivated at 75°C for 10 minutes with 5 mM EDTA. The total RNA was then purified by Phenol:Chloroform extraction. Total RNA (1 μg) was reverse transcribed using SuperScript III Reverse transcriptase (Life Technologies) and Oligo dT (Integrated DNA technologies). The qPCR was performed using KAPA SYBR FAST Universal 2x PCR Master Mix (KAPA Biosystems) on a Mastercycler EP Realplex real-time PCR system (Eppendorf) according to the manufacture’s instructions. Primers used in this study are:

eri-6/7 trans-spliced Forward 5’-tccaataatacgcttacgcatc-3’

eri-6/7 trans-spliced Reverse 5’-cttggagtgtcaatgattctgg-3’

**Data analysis**

**Comparison of averaged Pol II occupancy levels in the wild type and *hrde-1*(*tm1200*) mutant animals around the 5’ and 3’ ends of 3903 'H3K4me2/3-anchored’ genes** (**Figure 1A)**.

Libraries used for this analysis are 'PolII_S2_ChIP_WT', 'ChIP_input_WT', 'PolII_S2_ChIP_hrde1', 'ChIP_input_hrde1', 'PolII_8wg16_ChIP_WT', 'PolII_S5_ChIP_WT', 'PolII_8wg16_ChIP_hrde1', and 'PolII_S5_ChIP_hrde1'. 40-nt Illumina reads were aligned to the *C. elegans* genome (version WS190/ce6) using Bowtie (version 0.12.7) [[3](#_ENREF_3)]. 3903 'H3K4me2/3-anchored’ genes [[4](#_ENREF_4)] were used for this analysis. The zero position at the 5’ end of genes corresponds to the dyad position of the “plus-one” nucleosome. The zero position at the 3’ end of genes corresponds to the 3’ end of genes as annotated in the UCSC genome browser. For each gene, the ChIP-seq coverage at each position was calculated by counting the number of sequenced reads (extended as 200 nt sequence from the sequenced end). Both uniquely aligned reads and non-uniquely aligned reads were used for this analysis. The signals from non-uniquely aligned reads were normalized by the number of alignments (*e.g.,* if a read aligns to two different places in the genome, coverage of ½ was assigned at each place for this read). Metagene coverage was the sum of coverage on individual genes, normalized by the modeled DNA sized (200 nt), the number of genes, and sequencing depth (in millions).

**Pol II ChIP-seq analysis for the wild type and *hrde-1(tm1200)* mutant animals on 100,257 1-kb regions in the *C. elegans* genome** (**Figure 1B).**

1kb-coverage values were calculated by counting the number of sequenced reads aligned to each 1kb window in the *C. elegans* genome. The numbers were then normalized by sequencing depth (in millions). A 12-kb sequence at the end of chromosome I encodes *C. elegans* ribosomal RNAs. The actual copy numbers of the rRNA genes are larger than the copy numbers indicated by the annotated genome. This region was excluded for this analysis.

The 1kb-coverage values were used to identify GRTS (germline nuclear RNAi-mediated transcriptional silencing) regions. GRTS were defined as regions with at least 3-fold increase in Pol II ChIP-seq signal in the *hrde-1* mutant for all three sets of Pol II ChIP-seq experiments, excluding regions with 3-fold increase in ChIP input signals in the *hrde-1* mutant. To reduce false positives, we also required Pol II ChIP-seq signals in GRTS regions in the *hrde-1* mutant were above the mean value of the genome-wild 1kb coverage. GRTS with a minimal of 2-fold and 1.5-fold increase were similarly identified and listed in Supplementary Table S1.

**H3K9me3 ChIP-seq analysis for the wild type and *hrde-1(tm1200)* mutant animals on 100,257 1-kb regions in the *C. elegans* genome** (**Figure 2A).**

Libraries used for Fig. 2A and generated by this study were 'H3K9me3_ChIP_WT' and 'H3K9me3_ChIP_hrde1', 'PolII_S2_ChIP_WT', 'PolII_S2_ChIP_hrde1', 'PolII_8wg16_ChIP_WT', 'PolII_S5_ChIP_WT', 'PolII_8wg16_ChIP_hrde1', and 'PolII_S5_ChIP_hrde1'. Libraries used for Fig. 2A and generated by previous studies were 'H3K9me3_ChIP_WT_smg1_RNAi' (NCBI accession number: GSM855085) [[1](#_ENREF_1)], 'H3K9me3_ChIP_nrde2_smg1_RNAi' (NCBI accession number: GSM855086) [[1](#_ENREF_1)], and 'H3K9me3_ChIP_nrde4_smg1_RNAi' (NCBI accession number: GSM932876) [[5](#_ENREF_5)]. Whole genome alignments were described in Fig. 1A, except that 25-nt reads were used for aligning the three previously generated libraries. 1kb-coverage values were calculated as described in Fig. 1B. Regions with NRDE-2 and NRDE-4-dependent H3K9me3 were defined as regions with at least 3-fold decrease in H3K9me3 ChIP-seq signals in both *nrde-2* and *nrde-4* mutants when compared with the wild type, excluding regions with H3K9me3 ChIP-seq signals in the wild type that were below the mean value of the genome-wild 1kb coverage. Regions with HRDE-1*-*dependent H3K9me3 were defined using the same approach. GRH (germline nuclear RNAi-mediated heterochromatin) regions were defined as regions with NRDE-2, NRDE-4, and HRDE-1-dependent H3K9me3. GRH with a minimal of 2-fold and 1.5-fold decrease were similarly identified and listed in Supplementary Table S1.

**Proximity test for GRH and GRTS regions (Figure 2B)**. Percentage of the 215 kb GRH that were nearby a GRTS region was plotted as a function of the proximity cutoff (blue curve). The simulation (red curve) was done by randomly sampling 215 kb regions out of a total 100,000 kb regions. For each proximity cutoff value (0, 1, …, 99 kb), the frequencies that 0, 1, 2, …, S of the 215 randomly sampled regions that are within the proximity cutoff distance to a GRTS region were calculated using the binomial distribution. The frequencies for 0-S were added up to generate the accumulative frequency. The minimal number of S (Smin) that gave the accumulative frequency of at least 0.999999999999 was determined and plotted as a function of the proximity cutoff (red curve).

For RNA-seq analyses (small RNA, mRNA, or pre-mRNA), identical reads were collapsed as one read to avoid “jackpot” due to PCR amplification (Figure 3, 4, 5, and 6). Small RNA analysis in Figure 6A was performed using previously published small RNA-seq data: GSM996951 [[6](#_ENREF_6)] and GSM948669 [[7](#_ENREF_7)] for N2 and GSM996954 [[6](#_ENREF_6)] and GSM948670 [[7](#_ENREF_7)] for *prg-1*(*n4357*).

One biological replicate was performed for each of the ChIP-seq and RNA-seq experiments. We note that conclusions for GRTS were made by requiring changes to occur in all three sets of ChIP-seq experiments: Pol II (8WG16) ChIP-seq (N2 vs. *hrde-1*), Pol II (S2) ChIP-seq (N2 vs. *hrde-1*), and Pol II (S5) ChIP-seq (N2 vs. *hrde-1*). Similarly, conclusions for GRH were made by requiring changes to occur in all three sets of H3K9me3 ChIP-seq experiments: N2 vs. *hrde-1*, N2 vs. *nrde-2*, and N2 vs. *nrde-4*. Conclusions made about GRTS and GRH were further supported by pre-mRNA seq (N2 vs. *hrde-1*) and mRNA-seq (N2 vs. *hrde-1*).

**Reference:**

1. Gu SG, Pak J, Guang S, Maniar JM, Kennedy S, Fire A: **Amplification of siRNA in Caenorhabditis elegans generates a transgenerational sequence-targeted histone H3 lysine 9 methylation footprint**. *Nature genetics* 2012, **44**(2):157-164.

2. Parameswaran P, Jalili R, Tao L, Shokralla S, Gharizadeh B, Ronaghi M, Fire AZ: **A pyrosequencing-tailored nucleotide barcode design unveils opportunities for large-scale sample multiplexing**. *Nucleic acids research* 2007, **35**(19):e130.

3. Langmead B, Trapnell C, Pop M, Salzberg SL: **Ultrafast and memory-efficient alignment of short DNA sequences to the human genome**. *Genome biology* 2009, **10**(3):R25.

4. Gu SG, Fire A: **Partitioning the C. elegans genome by nucleosome modification, occupancy, and positioning**. *Chromosoma* 2010, **119**(1):73-87.

5. Buckley BA, Burkhart KB, Gu SG, Spracklin G, Kershner A, Fritz H, Kimble J, Fire A, Kennedy S: **A nuclear Argonaute promotes multigenerational epigenetic inheritance and germline immortality**. *Nature* 2012, **489**(7416):447-451.

6. Simon M, Sarkies P, Ikegami K, Doebley AL, Goldstein LD, Mitchell J, Sakaguchi A, Miska EA, Ahmed S: **Reduced Insulin/IGF-1 Signaling Restores Germ Cell Immortality to Caenorhabditis elegans Piwi Mutants**. *Cell reports* 2014, **7**(3):762-773.

7. Lee HC, Gu W, Shirayama M, Youngman E, Conte D, Jr., Mello CC: **C. elegans piRNAs mediate the genome-wide surveillance of germline transcripts**. *Cell* 2012, **150**(1):78-87.
